# Editorial: Polyphenols and betalains in obesity and metabolic syndrome

**DOI:** 10.3389/fnut.2025.1682319

**Published:** 2025-09-29

**Authors:** Victoria Ramírez, Carlos César Bravo-Reyna, Joyce Trujillo

**Affiliations:** ^1^Departamento de Nutrición Animal, Instituto Nacional de Ciencias Médicas y Nutrición Salvador Zubirán, Mexico City, México; ^2^Departamento de Cirugía Experimental, Instituto Nacional de Ciencias Médicas y Nutrición Salvador Zubirán, Mexico City, México; ^3^División de Materiales Avanzados, Instituto Potosino de Investigación Científica y Tecnológica (DMA-IPICYT), México y Secretaría de Ciencia, Humanidades, Tecnología e Innovación (SECIHTI), San Luis Potosí, México

**Keywords:** metabolic syndrome, polyphenols, betalains, flavones, antioxidants

As we all know, the prevalence of obesity and metabolic syndrome (MetS) is considered a global pandemic. One of its main consequences is the development of diabetes mellitus and all its comorbidities, including cardiovascular and chronic kidney disease. A global meta-analysis from 28 million people shows a 45% prevalence of MetS (1). Subsequently, multiple strategies have been implemented to reduce its prevalence, including pharmaceutical treatments and lifestyle modifications such as regular physical activity, a balanced diet, and stress management. However, many of these approaches have had only partial success due to the heterogeneity of each case and its underlying causes. Over the past decade, numerous researchers have been focused on identifying novel factors that may contribute to mitigating these effects in the population. Bioactive compounds, such as polyphenols, betalains, and isoflavones, extracted from plants, fruits, nuts, leaves, and roots, have been shown protective effects against obesity and metabolic disorders in experimental models and clinical trials. Although the molecular targets of these compounds are not yet fully described, they exhibit different biological activities, including antioxidant, anti-inflammatory, hypolipemic, and hypoglycemic, among others (2).

In this Research Topic, the primary focus was to investigate how polyphenols and betalains from functional foods can modulate metabolism to improve specific biochemical markers and prevent the deleterious effects of MetS, employing both basic and clinical approaches.

In this sense, a study conducted in an obesity model fed with 1.5% betaine (found in beetroot) demonstrated improved lipid metabolism. Betaine supplementation has been shown to significantly reduce glycemia, triglycerides, cholesterol, and adipocyte size. Additionally, omics analyses revealed changes in the microbiome, lipidome, and transcriptome. Betaine restores the balance of the Firmicutes/Bacteroidetes ratio, increases beneficial bacteria, such as Lactobacillus, which is associated with improved metabolism, and restores levels of Short Chain Fatty Acids related to an anti-inflammatory state. Furthermore, it enhanced lipid oxidation pathways, increasing metabolites like Acetyl-CoA and Pnlp3 among others, which showed the importance of bioactive molecules in our daily diet may improve the general metabolism (Wang et al.). Another study investigated the role of polyphenols from Peonies in a rodent model of diabetes. The authors demonstrate that peony extract improves glycemia and insulin resistance, reduces hyperlipidemia, and reduces liver and kidney index. It can also restore the oxidative environment by increasing glutathione peroxidase activity and reducing lipoperoxidation, as well as partially restoring fatty acid profile. The authors also showed that peony polyphenols may improve gut dysbiosis by increasing *Bacteroidetes* and *Firmicutes* and by reducing *Proteobacteria*. These changes were associated with improved amino acid metabolism and overall metabolic status in the diabetic model (Chen et al.).

As we mentioned before obesity is one of the most common causes of MetS. Lu et al. demonstrate in an experimental rodent model of obesity that quercetin, a flavone with biological activity, restores the levels of 3-indolepropionic acid, which is related to liver health, by modulating lipid metabolism. Animal feed with quercetin presented lower body weight, liver weight, adipocyte size, fat percentage, also quercetin was able to reduce pro-inflammatory markers in adipose tissue, liver and colon, with may improve the adipocyte disfunction with may result in a better metabolism signaling, once again showing that functional foods may have an essential role in health and disease. To improve polyphenol extraction from plants, such as *Lonicera Japonica*, known for its glucosidase activity, Li et al. proved an ultrasound-assisted extraction method, the results show an efficient process with a 9% yield, with high concentrations of other phenols and biomolecules with potential biological activity. The authors also show that the polyphenols possess glucosidase activity, which potentially can be used as hypoglycemic coadjuvants. Another interesting study, conducted by Nsor et al. used *Annona muricata* (soursop) extract, rich in saponins, flavones, tannins, anthocyanins, and phenols, which reduces blood pressure in induced L-NAME hypertensive rats. The results show that soursop extract improved lipid metabolism, reduced oxidative stress in aorta heart and kidney and increase antioxidant response in dose dependent manner, the authors suggest that the extract poses anti-inflammatory properties and may reduce the atherogenic risk in hypertensive subjects. Additionally, several clinical trials aimed to prove the benefits of bioactive molecules. An observational cross-sectional study examined the consumption of flavones in children and adolescents using data from NHANES and other databases, including anthropometric indexes. Specific compounds identified included eriodyctiol, hesperetin, and naringenin. Multivariate analysis revealed that higher flavone levels were associated with a lower risk of overweight and obesity, regardless of gender (Liu et al.). A similar result has been reported by others (3).

In conclusion, all this evidence suggests that consuming food rich in bioactive molecules, such as polyphenols, flavonoids, and anthocyanins, may reduce the risk of developing metabolic diseases, mitigate the risk of comorbidities, and even lower the risk of mortality. All this suggests that efforts should be directed to personalized nutrition with a multidisciplinary focus to reduce the prevalence of MetS.
